# Epstein-Barr virus-associated multiple sclerosis: recent mechanistic advances and clinical therapeutic perspectives

**DOI:** 10.3389/fimmu.2026.1799161

**Published:** 2026-05-21

**Authors:** Ruogu Cheng, Ye Gao, Ruoyi Zheng, Muzi Wen, Gaoling Li, Wenbin Pan, Yuqiao Liao, Linxin Wen, Yueyang Hu, Hannah Zhao-Fleming, Pei Shang

**Affiliations:** 1 Department of Neurology, Nanfang Hospital, Southern Medical University, Guangzhou, China; 2 Department of Neurology, Southern California Permanente Medical Group, Alton/Sand Canyon Medical Office 2, Irvine, CA, United States; 3 Department of Neurology, Mayo Clinic College of Medicine, Rochester, MN, United States

**Keywords:** autoimmune response, EBV-targeted therapy, Epstein- Barr virus, multiple sclerosis, neuroinflammation

## Abstract

Multiple sclerosis (MS) is an immune-mediated chronic inflammatory and degenerative disease of the central nervous system (CNS). Typically occurring in young and middle-aged individuals, untreated MS can have high rates of disability and recurrence, thereby imposing a significant burden on the patient, their family, and society. Many factors are implicated in the etiology of MS, with the relationship between Epstein-Barr Virus (EBV) infection and the development of MS being the subject of extensive research recently. When the human body experiences a decline in immune function, it may trigger reactivation of EBV, and this reactivation is also believed to increase the risk of onset or relapse of MS. Currently, the phenomenon of cross-reactivity resulting from molecular mimicry following EBV infection (including reactivation status) is theorized as a contributing etiology. EBV-mediated abnormalities in T cells and B cells also play a key role in the development of MS. However, the underlying mechanisms have not been thoroughly understood. Meanwhile, the limited availability of effective treatment options for MS, in particular MS progression, underscores the urgent need for novel therapeutic strategies. Here, we discuss the pathophysiological mechanisms underlying MS, specifically emphasizing the relationship between EBV infection and the disease pathology. Furthermore, we introduced relevant pharmacological targets in order to propose a broader range of therapeutic alternatives for individuals diagnosed with MS.

## 1 Introduction

MS, the most prevalent leading cause of non-traumatic neurologic disability affecting young adults, is categorized into two clinical phases that are not mutually exclusive: relapsing and progressive. MS is often subcategorized: Radiographically isolated syndrome (RIS), Clinically Isolated Syndrome (CIS), Relapsing-Remitting Multiple Sclerosis (RRMS), Secondary Progressive Multiple Sclerosis (SPMS), and Primary Progressive Multiple Sclerosis (PPMS). Most MS cases typically begin with a relapsing-remitting course, with some patients developing a progressive phase, a gradual deterioration of neurological function that is separate from clinically obvious relapses. Studies have shown that people around the age of 20–40 are more predisposed to the first-episode MS with a female predominance. However, the sex ratio in the case series of the early 1900s was nearly equal before the majority of developed nations experienced a consistent rise in the sex ratio, reaching nearly 3 females to every 1 male (1). One potential contribution to this phenomenon is that cigarette smoking contributes approximately 50% to the 40% higher incidence of MS in women (2). Moreover, studies of genome-wide associations have revealed over 150 single-nucleotide polymorphisms linked to the risk of MS (3). Along with a person’s genetic background (encoding HLA-DR15), EBV infection, ultraviolet B (UVB) exposure, smoking, and vitamin D deficiency, all have a part in the causative chain that leads to the development of MS (4).

Among these factors, the infection caused by EBV seems to be fundamentally associated with MS pathophysiology. EBV, a double-stranded DNA virus, is a member of the γ herpes virus family. First identified in Burkitt lymphoma in 1964, it was the earliest virus known to cause cancer (5, 6). The general population exhibits a high susceptibility to EBV, which infects more than 90-95% of adults globally (7). The life cycle primarily encompasses three stages: the initial infection, the incubation period, and the subsequent lytic reactivation. A comprehensive study involved over 10 million military personnel in the United States, revealing a 32-fold increase in the risk of developing MS following infection with EBV (8). Notably, among the 801 individuals who remained EBV-seronegative throughout the study period, none developed MS (8). Previous studies have demonstrated that serum neurofilament light (sNfL), a sensitive biological marker of neuronal damage, increased 6 years before the onset of clinical MS (9). In this study, EBV infection was earlier than the sNfL rise, before the onset of MS. This suggested a potential causal relationship between EBV infection and the onset of MS.

The underlying mechanism of EBV in MS pathogenesis is still unclear, despite a number of related studies. In this review, we mainly focused on EBV infection and MS, and tried to illustrate the potential pathogenic mechanisms and suggest relevant treatments based on current and past knowledge.

## 2 Characteristics and life cycle of EBV

EBV, a double-stranded DNA virus, is a member of the γ herpes virus family and has an infection prevalence of approximately 90-95% among adults globally (10). First identified in Burkitt lymphoma in 1964, EBV is the earliest virus known to cause cancer in human and can establish a lifelong latency in the body (5, 6). Analyses showed that EBV comprises genomic capsids and enveloping structures. The DNA genome is encapsulated within an icosahedral nucleocapsid and includes approximately 80 open reading frames. These include nuclear antigens such as EBNA1, EBNA2, EBNA3A, EBNA3B, EBNA3C, and EBNA-L, as well as membrane proteins like LMP1 and LMP2, in addition to 44 non-coding RNAs (5, 11). EBNA and LMP1 are uniformly expressed in human cells, and LMP1, EBNA2, and EBNA3C play a significant role in EBV immortalizing B cells. Of note, two isoforms of the EBV genome, namely type 1 and type 2, exhibit polymorphisms in the genes encoding EBNA2 and EBNA3 (12). Evidence indicated that in comparison to type 2, type 1 is more likely to induce the transformation and immortalization of B lymphocytes *in vitro*, resulting in a higher detection rate for associated diseases (13).

**Figure 1:**
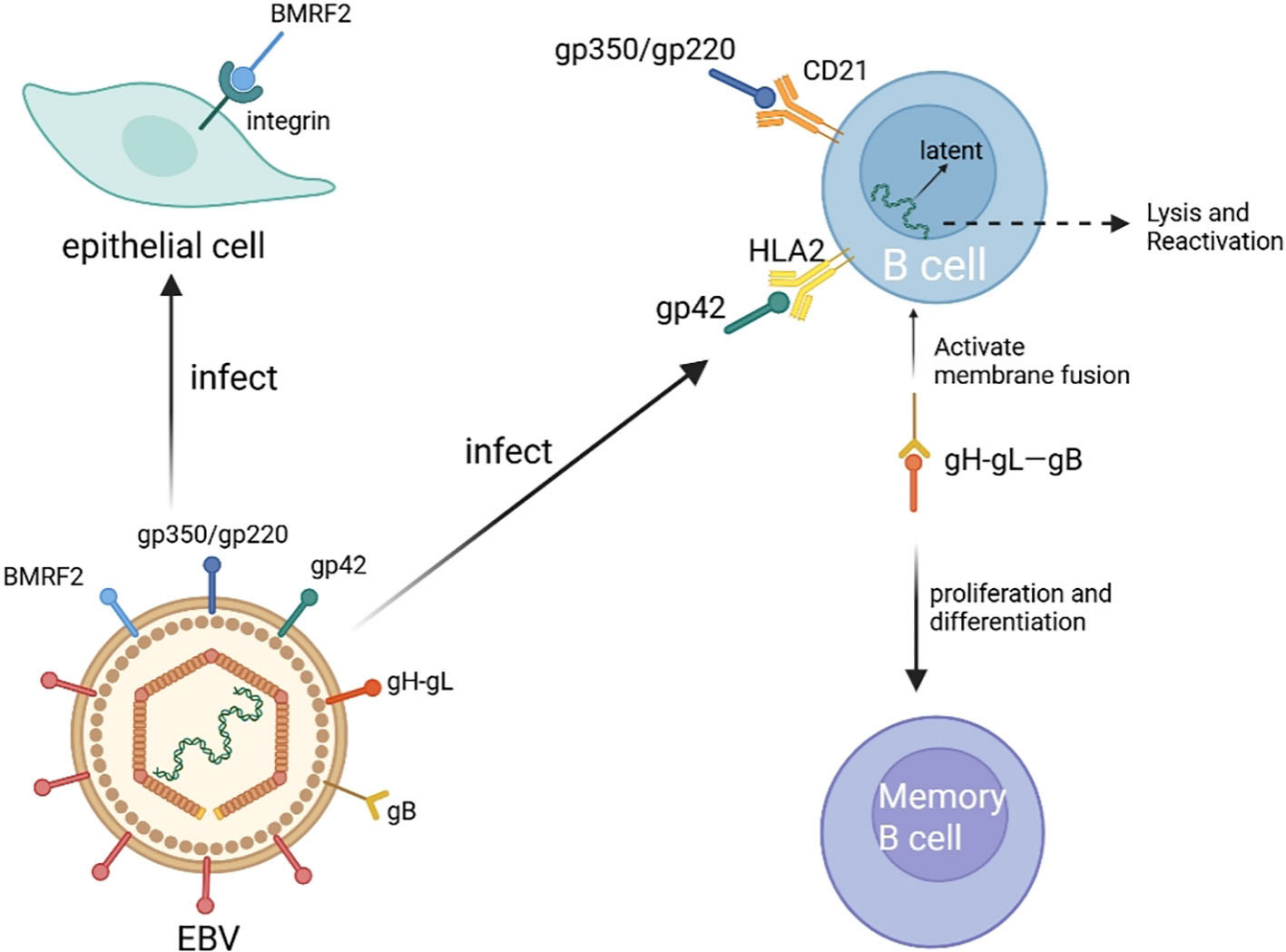
Epidemiology and life cycle of EBV. The EBV envelope contains glycoproteins like gp350 and gp42. Gp350/220 binds to CD21 on B cells, facilitating attachment. Gp42 interacts with HLA II and forms a complex with gH-gL, then engages gB to activate membrane fusion. EBV then enters a latent state in B cells, promoting their proliferation into memory B cells, which serve as a reservoir. Gp350 and gp42 presence is due to EBV BMRF2 binding to integrins. This figure is illustrated by Biorender.

The envelope of EBV contains virus-encoded membrane glycoproteins, such as gp350 and gp42 (14). The initial infection with EBV is typically spread via saliva (5). Through gp350/gp220, EBV can recognize and bind to complement receptor CR2 (CD21), expressed on B cells, thereby facilitating its attachment to host B cells (15). Subsequently, gp42 interacts with human leukocyte antigen II (HLA II), forming a stable complex with gH-gL, and then engages with the signaling receptor gB to activate membrane fusion with B cells (14, 16). In the following stages, EBV remains predominantly in a latent state within B cells. It promotes the proliferation and differentiation of these B cells, leading to their transformation into memory B cells, which serve as a reservoir for the virus’s long-term persistence (17). Under certain circumstances, the cleavage and reactivation of EBV may take place, which may trigger an immune-mediated response that leads to the maintenance or occurrence of certain diseases (18) or an increased risk of cancer (19). Nevertheless, gp350 and gp42 are not essential for this process; rather, their presence is a consequence of the binding of EBV BMRF2 to integrins (20) (**Figure 1**).

The general population exhibits a high susceptibility to EBV, which infects more than 90-95% of adults globally (7). The life cycle primarily encompasses three stages: the initial infection, the incubation period, and the subsequent lytic reactivation. It seems that primary infection with EBV typically occurs in childhood, adolescence, or early adulthood, after which a lifelong incubation period is established without obvious symptoms in healthy individuals (21). In adolescents and young adults, individuals infected with EBV may exhibit symptoms of infectious mononucleosis (IM) (22), a more severe infection characterized by marked fatigue, fever, and swollen lymph nodes. There is considerable evidence on the risk associated with IM and subsequent MS onset. Patients with a history of IM had a double risk of developing MS compared to people without a history of IM (23), with a stronger association in patients who develop IM in adolescence rather than later in life (24, 25). Additionally, a compromised immune system may result in patients experiencing reinfection with EBV or the lysis and reactivation of EBV (26). This may contribute to the development of certain malignancies. One of the key mechanisms of this oncogenic process is the immortalization of infected cells, particularly B cells. This oncogenic process is driven by the expression of latent viral proteins such as EBNA2 and LMP1, which promote cell proliferation and inhibit apoptosis (27).

EBV infection has also been linked to a range of immune-mediated disorders, including Sjögren’s syndrome (SS), systemic lupus erythematosus (SLE), rheumatoid arthritis (RA), MS, and so forth (28–32). Studies indicate that patients with these diseases have evidence of EBV infection and increased EBNA1-specific antibody titers, and that the onset of autoimmune diseases can often occur long after the initial EBV infection (33, 34).

### 2.1 Epidemiology of MS associated with EBV

Recent studies have indicated a possible link between EBV and the onset of MS. A study monitored data from more than 10 million U.S. military recruits and documented EBV infection in 955 patients with MS (pwMS). The results showed a 32-fold increased risk of developing MS after EBV infection, with EBV infection occurring earlier than the detection of sNfL, before the onset of MS (8). Notably, among the 801 individuals who remained EBV-seronegative throughout the study period, none developed MS (8). This supports a causal relationship between EBV infection and the onset of MS, although further studies are needed.

In 2001, a prospective study demonstrated that a notable elevation in serum anti-EBV antibody titers occurred before the onset of MS (35). In a cohort of 325 pwMS, the positivity rate for EBNA1 was found to be 97.8% (318/325), while the viral capsid antigen (VCA) positivity rate was 99.7% (324/325). Notably, all pwMS exhibited positivity for either EBNA1 or VCA (36). In addition, Sun et al. extracted blood-brain barrier (BBB) biomarkers from MRC IEU Open GWAS data infrastructure and confirmed a close relationship between anti-EBNA IgG levels and MS risk (OR=225.62, P=5.63E-208) and disease severity (OR=1.11, P=0.04) by MR analysis (37). Elevated anti-EBV titers may constitute an early link in the pathogenesis of MS. Gerald et al. demonstrated that in blood samples collected 30 years before the onset of MS, the titers of the EBNA complex and its component EBNA-1 antibodies were significantly increased in pwMS. This phenomenon precedes the onset of symptoms by 15–20 years and persists thereafter (38).

The distribution of MS subtypes varies with age at onset. In pediatric MS, RRMS accounts for more than 95% of cases (39). In contrast, among adult MS patients, RRMS remains the most common subtype, accounting for approximately 85-90% of cases, while PPMS accounts for only 10-15% of adult cases (40). Meanwhile, the mean age at onset of PPMS is approximately 40 years, later than that of RRMS. Therefore, the proportion of PPMS among MS patients increases with age (41, 42). A difference between adults and children with MS is that children have a lower ratio of men to women with MS. Also, the ratio of male to female children under 10 years of age is lower than that of children over 10 years of age (P=0.03) (43). Moreover, for adults, women are more likely than men to develop MS. However, the sex ratio in the case series of the early 1900s was nearly equal before the majority of developed nations experienced a consistent rise in the sex ratio, reaching nearly 3 females to every 1 male (1).

However, despite the presence of anti-EBV antibodies in serum among nearly all patients, only a minority of individuals infected with EBV go on to develop MS (44). This suggests that the onset of MS may result from a combination of viral infections, genetic predisposition, immune status, and environmental factors (such as reduced sunlight exposure, vitamin D deficiency, and smoking) (4, 45, 46). Meanwhile, we noted that the strength of the association between different viruses and MS also varies significantly. In addition to EBV, cytomegalovirus (CMV) and varicella-zoster virus (VZV) have also been included in related studies. Studies have found that the CMV infection rate in pwMS (58.7%) is lower than that in the general population (40%–90%), and although their serum CMV IgG levels are slightly higher than those of healthy controls, the difference is not statistically significant (47). At the same time, the immune evasion characteristics of CMV may partially alleviate autoimmune responses and pro-inflammatory environments, contrasting with the pathogenic effects of EBV (48) (**Table 1**). The infection rates of VZV are similar in MS patients and healthy populations (97.5% and 98.5%, respectively), and there is no significant difference in VZV IgG levels between the two. Its association with MS is not yet clear, though a potential link may exist in some non-European regions (49) (**Table 1**). In contrast, the association between EBV and MS is both widespread and strong, with nearly all pwMS having been infected with EBV and showing significantly elevated levels of anti-EBV antibodies, further supporting the central role of EBV in the pathogenesis of MS. Therefore, the occurrence of MS due to EBV infection may be attributed to specific and distinct mechanisms.

**Table 1:** Comparison of the correlation between different viral infections and MS.

Virus	Infection	Relevance to MS	Antibody levels
CMV	The infection rate in MS patients is 58.7%. The CMV seropositivity rate varies between 40%~90% in the total population, and pwMS have a lower rate of CMV seropositivity compared to HCs (47, 133).	Shorter leukocyte telomere length was observed in MS patients compared to non-MS controls and was associated with disability status and progression. On the other hand, the immune evasion of CMV may alleviate autoimmune responses and a pro-inflammatory environment (134–136).	CMV IgG levels were higher in MS patients, but the difference was not significant. At the same time, the positivity rate of CMV IgG is lower than that of EBV (49, 135).
EBV	The infection rate is nearly 100% in MS patients and exceeds 95% in healthy individuals (8, 36).	There is a strong association, and almost all MS patients have been infected with EBV (8, 38).	The levels of EBV VCA IgG and EBNA-1 IgG in MS patients were significantly higher than those in HCs (38, 49).
VZV	The infection rate was higher in MS patients and healthy controls, 97.5% and 98.5%, respectively (49)	The association is unclear, but it may be associated with the occurrence of MS in some non-European regions (49)	There was no significant difference in VZV IgG levels between MS patients and HCs (49)

CMV, Cytomegalovirus; EBV, Epstein-Barr Virus; HC, Healthy Control; HLA, Human Leukocyte Antigen; IM, Infectious Mononucleosis; MS, Multiple Sclerosis; VZV, Varicella-zoster Virus.

Additionally, the risk of developing MS after EBV infection may not be uniform across all infected individuals, and emerging evidence suggests that the age at primary infection is an important modifier of this risk. The age at primary EBV infection may critically influence the risk of MS. In the United States, nationally representative data showed that EBV seroprevalence among children aged 6–19 was substantially lower in Non-Hispanic Whites compared to Mexican-Americans and Non-Hispanic Blacks. Despite their lower seroprevalence, Non-Hispanic Whites have a higher MS incidence, whereas Mexican-Americans and Non-Hispanic Blacks, who were infected with EBV earlier in childhood, had a lower risk of MS (50). This paradox supported the “delayed infection hypothesis”, which posited that early EBV exposure in childhood may be protective, whereas delayed primary infection, typically manifesting as infectious mononucleosis in adolescence, increased the risk of MS (51, 52).

## 3 Potential mechanisms

EBV is considered a potential trigger for MS. Nevertheless, the mechanisms underlying this association remain unclear. In this review, we presented various lines of evidence indicating that EBV significantly contributes to the pathogenesis of MS. To provide a deeper understanding, we now discuss the molecular and cellular details of each proposed mechanism in the following subsections.

### 3.1 Molecular mimicry

**Figure 2:**
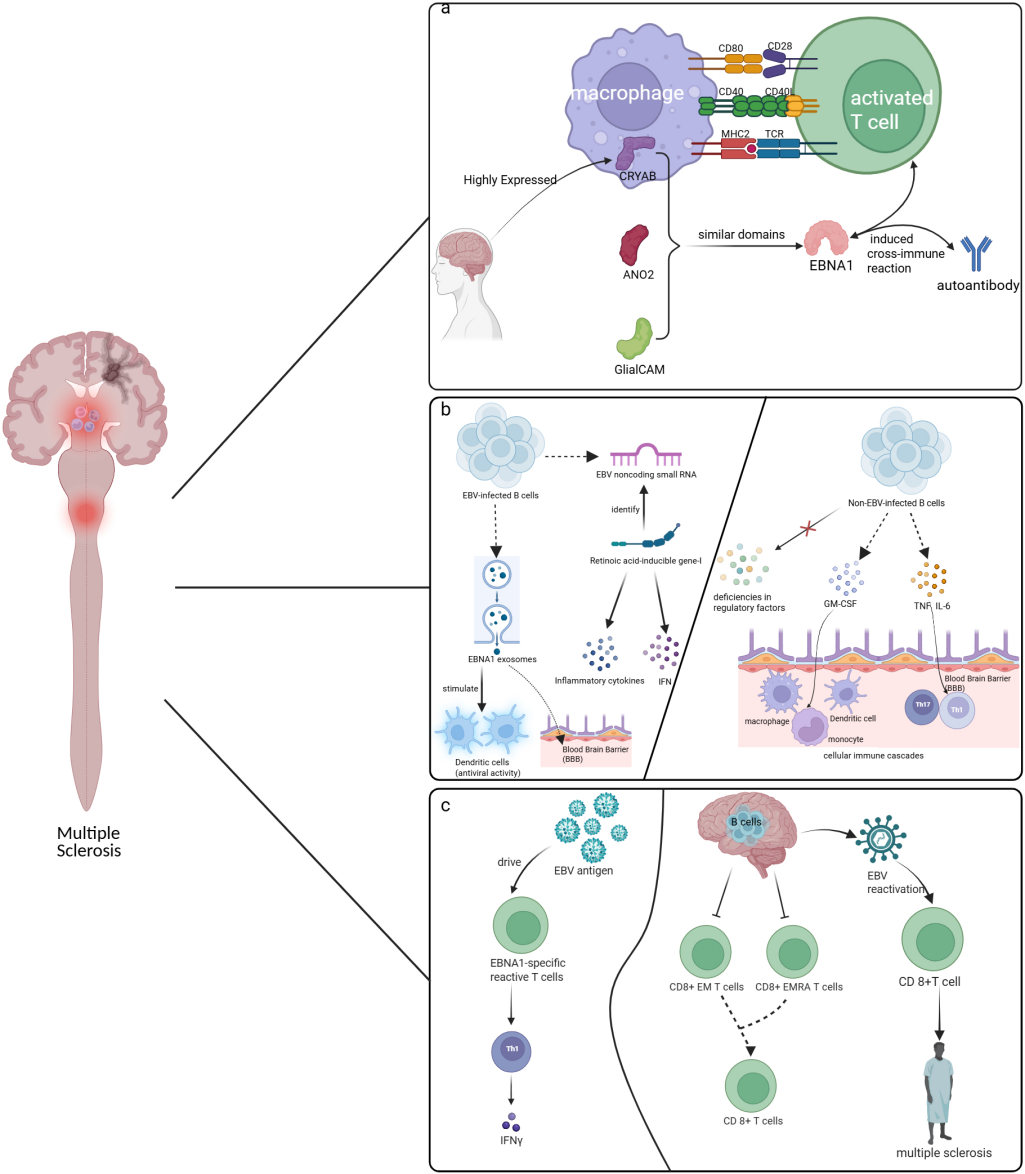
Molecular mimicry between microbial and neural antigens. EBNA1 fragments in CSF structurally mimic CNS ion channel ANO2, suggesting a role in MS via molecular mimicry. In early MS, CRYAB-loaded macrophages express MHC II/CD40/CD80 to activate T cells. EBNA1–CRYAB structural similarity triggers cross-reactive antibodies and T cells against myelin. GlialCAM may also mimic EBNA1, further driving CNS autoimmunity. **(a)** EBNA1 fragments in CSF structurally mimic CNS ion channel ANO2, suggesting a role in MS via molecular mimicry. In early MS, CRYAB-loaded macrophages express MHC II/CD40/CD80 to activate T cells. EBNA1–CRYAB structural similarity triggers cross-reactive antibodies and T cells against myelin. GlialCAM may also mimic EBNA1, further driving CNS autoimmunity. **(b)** MS patients exhibit a broader, higher-affinity EBNA1-specific TCR repertoire. These Th1-biased T cells secrete abundant IFN-g. Concurrently, EBV-specific CD8^+^ T cell immunity is compromised. EM and EMRA effector subsets are reduced and further decline with age, while naive CD8^+^ T cells also wane. This impaired surveillance permits EBV reactivation within the CNS, triggering aberrant CD8^+^ T cell expansion against viral antigens and intensifying autoimmune damage that drives MS progression. **(c)** MS patients exhibit a broader, higher-affinity EBNA1-specific TCR repertoire. These Th1-biased T cells secrete abundant IFN-γ. Concurrently, EBV-specific CD8^+^ T cell immunity is compromised, permitting EBV reactivation within the CNS. This triggers aberrant CD8^+^ T cell expansion against viral antigens, intensifying autoimmune damage that drives MS progression. This figure is illustrated by Biorender.

Molecular mimicry between microbial and neural antigens is hypothesized to be a part of MS pathogenesis. In some studies, certain EBV antigens, especially EBNA1, have been reported as targets of self-produced antibodies in pwMS, potentially promoting cross-immune responses. Autoimmune antibodies identify numerous domains of EBNA1, and these specific domains allow antibodies and monocytes triggered by viral infection to cross-react with host proteins due to molecular mimicry (48) (**Figure 2a**).

ANO2, a protein implicated in the electrical conduction within axons, has previously been recognized as an autoimmune target in the context of MS (53). Nonetheless, it remains unclear whether it is a primary immune response in the pathophysiology of MS or a secondary immune response to persistent inflammation (54). In 2019, Tengvall K et al. showed that a fragment of EBNA1 in human cerebrospinal fluid (CSF) has structural similarities to the ion channel ANO2 expressed in the central nervous system (55). This suggests that molecular mimicry of ion channels in the CNS may contribute to the development of MS. Moreover, recent studies have offered additional mechanistic evidence indicating that ANO2-specific T cells play a role in the development of MS. Thomas et al. found that ANO2-specific CD4 T cells are more common in pwMS, and these T cells can simultaneously recognize EBNA1 and ANO2. Furthermore, ANO2 pre-immunization can exacerbate the disease in the EAE mouse model (56). These findings provide direct T cell-level evidence that EBV infection may participate in MS pathogenesis through ANO2-mediated molecular mimicry.

Crystallin Alpha B (CRYAB) is highly expressed in the brains of pwMS. In early MS, CRYAB-loaded macrophages can express MHC II, CD40, and CD80, which stimulate T cell responses, demonstrating their potential for myelin immunogenicity (57, 58). Studies have attempted to elucidate the involvement of CRYAB in the pathogenesis of MS associated with EBV. The results indicate that in EBV-infected MS, the structural similarity between CRYAB and EBNA1 leads to the activation of pertinent autoimmune antibodies and reactive T cells. This phenomenon facilitates the cross-reactivity of T cells with both microbial sequences and peptide sequences derived from myelin (59, 60).

In the development of MS, GlialCAM has also been proposed and may engage in molecular mimicry mechanisms alongside EBNA1 (61–63). Additionally, other alternative mechanisms have been elucidated. This may be due to the interaction between the GlialCAM-specific cells and the NKG2A receptor present on the surface of natural killer (NK) cells, which subsequently inhibits the cytotoxic activity of NK cells and allows for evasion of immune surveillance. Furthermore, it can also affect the proliferation and activation of T cells (63).

Among the three aforementioned EBNA1 targets, the EBNA1-GlialCAM axis is supported by the most robust evidence to date. This phenomenon is primarily attributable to the research conducted by Lanz et al., which furnished direct evidence from target organs demonstrating that clonally expanded B cells within the cerebrospinal fluid of pwMS are capable of concurrently binding to both EBNA1 and GlialCAM (61). Furthermore, this finding has been corroborated through extensive cohort studies and substantiated by structural biology analyses (62, 64). In contrast, while the evidence for ANO2 includes, in addition to serological data, expression evidence in MS brain tissue and functional validation of T cell cross-reactivity (55, 56), its clonal B cell evidence in the cerebrospinal fluid is not as direct as that of GlialCAM. Meanwhile, although CRYAB is supported by T cell cross-reactivity data (59), the evidence linking CRYAB-directed immunity to EBV infection is relatively limited. Thus, although all three targets may contribute to MS pathogenesis, GlialCAM currently has the most direct and compelling evidence. Whether the other targets act as primary drivers of pathogenesis or represent secondary phenomena remains to be further elucidated in future studies.

### 3.2 Inflammatory cytokines against EBV

The impact of the inflammatory response to EBV on the pathogenesis of MS has been widely discussed. EBV-infected cells that remain in a latent state *in vivo* can synthesize EBV-encoded small RNAs (EBERs) (65). These EBERs can be identified by *Retinoic acid-inducible gene-I (RIG-I)* and activate their downstream signaling, allowing genes for type I interferons (IFNs) and other IFNs to be induced in EBV-infected B cells (65, 66). Furthermore, the activation of *RIG-I* has the potential to induce the synthesis of inflammatory cytokines. The exosomes associated with EBNA1 facilitate the activation of the inflammatory response in dendritic cells (DCs) against EBV (67). Under specific circumstances, exosomes may traverse the BBB by penetrating brain microvascular endothelial cells (BMECs) (68).

On the other hand, non-EBV-infected B cells also have inflammatory features. B cells primarily produce interleukin-6 (IL-6), granulocyte-macrophage colony-stimulating factor (GM-CSF), lymphotoxin a (LT-α), tumor necrosis factor (TNF), and various chemokines. These factors are characterized by elevated concentrations and diminished expression of regulatory factors (69–71). Given that many of the same cytokines are involved in the pathogenesis of typical MS, they may contribute significantly to the disease’s development and progression (72–74). TNF and IL-6 can elicit dysregulated responses in Th17 and Th1 cells. Additionally, they promote a pro-inflammatory response in myeloid cells through the action of GM-CSF, thereby exacerbating the immune cascade associated with relapse (71) (**Figure 2b**).

### 3.3 Dysregulation of T cells’ immune control

The immune regulation of EBV infection exhibits dysregulation at the level of T cells. Previous studies have found that immune dysregulation can be observed in EBV-related diseases. Prior to the manifestation of symptoms in patients diagnosed with IM, no discernible abnormalities were observed in CD4+ and CD8+ T lymphocyte populations. However, a notable increase in the activation of CD8+ T cells was recorded following the onset of symptoms (75). In addition, there is a broader repertoire of EBV-specific TCRs in pwMS. EBNA1-specific reactive T cells in pwMS recognize a wider range of EBNA1 epitopes and have a higher affinity, resulting in enhanced responsiveness to EBNA1 (76). Notably, these cells are primarily CD4+ T cells, which serve as the principal mediators of immune control specific to EBV in individuals who are virus carriers (77). These CD4+ T cells operate as Th1 cells and are capable of producing elevated levels of IFNγ (78).

Previous studies have indicated that the capacity of CD8+ T cells to effectively target EBV in pwMS is impaired and deteriorates with advancing age (79). The numbers of CD8 effector memory T cell (EM) and effector memory redistribution T cell (EMRA) subsets in pwMS were significantly reduced, leading to an inadequate immune response (80). Notably, the patients included in this study had not received glucocorticoids or immunosuppressive agents for at least three months. As individuals age, there is a further decline in the population of naïve CD8 T cells, which intensifies the reduction of effector EM and effector memory re-expressing EMRA subsets and thus contributes to a detrimental cycle of immunological control deficiencies (80). If EBV does establish latency within the CNS, this impairment of the immune system may result in the inadequate elimination of the infection, thereby allowing periodic reactivation of the virus. However, it should be noted that evidence for EBV latency in the brain is not universally consistent, and this remains a debated finding (81).

In addition, another hypothesis also links EBV to the pathogenesis of MS, focusing on newly synthesized EBV antigens after viral reactivation. These viral antigens are suggested to drive an aberrant, and potentially ineffective, expansion of specific CD8+ T cells. Rather than clearing the infection, this chronic antigenic stimulation is thought to contribute to a pro-inflammatory environment within the central nervous system, which could, in theory, potentiate the autoimmune damage seen in MS (82) (**Figure 2c**). The precise role of these T-cell responses and their direct contribution to disease progression remains an active area of investigation.

Considering the correlation between EBV infection and the onset of MS, it has also been posited that the onset of MS may result from insufficient regulation of the immune response mediated by EBNA1 (63).

Clinical research has confirmed that EBNA1-specific IgG antibodies are frequently detectable in the CSF of pwMS (83), and there is a view that the titer of EBNA1 antibodies is associated with the risk of MS onset. However, it is noteworthy that although elevated EBNA1 levels are correlated with the initial onset of MS, not all individuals with high EBNA1 expression will progress to MS. This phenomenon is mainly related to the presence of specific NKG2C+ and NKG2D+ cell subsets in pwMS, which may influence disease progression by regulating immune responses (63).

### 3.4 The interaction between EBV and HLA

HLA - DR15 is considered to be one of the most critical genetic risk factors associated with MS (84). The combined influence of the immune response triggered by EBV infection and the presence of the HLA - DR15 gene substantially elevates the risk of developing MS, with an estimated increase of at least sevenfold (85).

**Figure 3:**
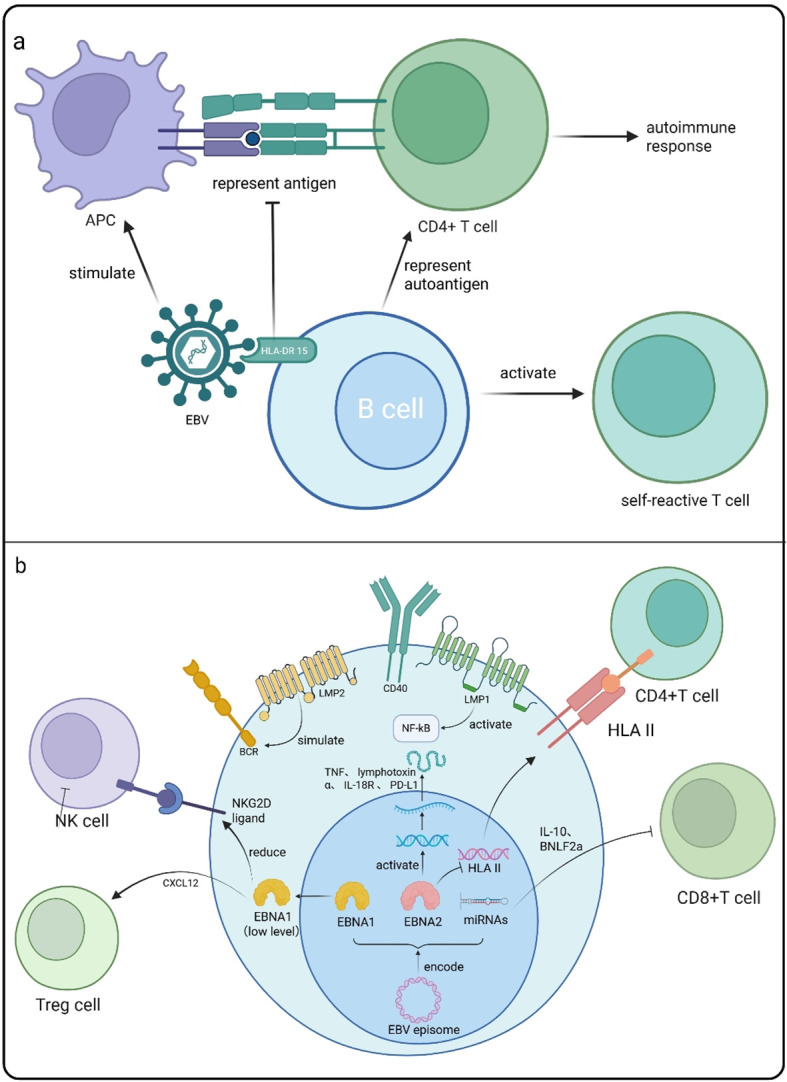
**(a)** Latent EBV-infected B cells express abundant EBERs, which RIG-I recognizes to trigger type I IFN and pro-inflammatory cytokine production. EBNA1-containing exosomes released from these B cells activate DCs and can cross the BBB, potentially seeding the CNS. Independently, non-infected B cells secrete high levels of IL-6, GM-CSF, TNF, and chemokines, which skew Th17/Th1 responses and amplify myeloid-mediated inflammation, driving MS relapse. **(b)** HLA-DR15 restricts EBV-peptide presentation by APCs, fostering EBV persistence in B cells. Within the CNS, these infected B cells present both viral and self antigens to CD4^+^ T cells, amplifying autoimmunity. Also, these B cells can activate autoimmune T cells, thereby promoting the progression of MS. EBV glycoprotein epitopes are selectively enriched for HLA-DR15 binding, and HLA-DR15 homozygotes mount markedly stronger T-cell responses to these epitopes than non-carriers. EBV-encoded proteins LMP1, LMP2, EBNA1, and EBNA2, along with EBV miRNAs, collectively promote B cell survival and evade immune surveillance. LMP1 mimics CD40 to activate NF-kB, driving B cell proliferation and survival. LMP2 simulates BCR signaling to bypass T cell support, maintaining latency. EBNA1 stimulates CXCL12 and downregulates NKG2D ligands to evade NK cells. EBNA2 activates immune regulatory genes (e.g., TNF, PD-L1) while suppressing interferon response genes and HLA II. EBV miRNAs target host immune genes, dampening CD8+ T cell responses. This figure is illustrated by Biorender.

Antigen-presenting cells (APCs) possess the capability to present peptides to CD4+ T cells, augmenting the immune response. HLA is expressed on APCs, affecting the processing and presentation of APC surface antigens, and hindering the presentation of EBV antigens to CD4+ T cells, resulting in the accumulation of EBV in B cells (84, 86, 87). In the CNS, EBV-infected B cells can present their own antigens to CD4+ T cells in response to EBV, triggering an immune response (88). Additionally, Drosu et al. reported the specific enrichment of EBV glycoproteins in epitopes associated with HLA-DR15. Further comparisons found that HLA-DR15 homozygous individuals had markedly heightened T cell responses to viral glycoprotein compared to control subjects who do not possess the HLA-DR15 allele (89) (**Figure 3a**). Apart from this, some other HLA phenotypes are also related to the pathogenesis of MS caused by EBV, which we have shown in **Table 2**.

**Table 2:** Association between different HLA phenotypes and EBV-related MS.

Phenotype	Mechanisms	Findings
HLA-E*01:03	Provides protection through effective virus clearance. High expression alleles can induce strong EBV BZLF1-specific HLA-E-restricted CD8^+^ T cell responses, effectively inhibiting virus transmission (63).	• Has a protective effect on IM and MS. • Homozygous genotype provides the strongest protective effect.
HLA-E*01:01	HLA-E can present EBV peptides such as BZLF1, be recognized by CD8 bright cells, and participate in the immune regulation of EBV infection (137). At the same time, the HLA-E*01:01 allele exhibits low expression levels, which may lead to immune regulation failure and reduced ability to control EBV-infected B cells (63), thereby increasing the risk of MS in individuals who develop a strong immune response after EBV infection (138).	• There is a significant additive interaction with a history of IM. • No significant association between individuals without an IM history and MS risk.
HLA-A*02	Provide protection through effective virus removal. HLA-A*02 can present immunodominant EBV peptides, inducing a strong CD8^+^ T cell response and effectively clearing EBV-infected B cells (139). This allele is less frequent in MS patients compared with healthy controls, suggesting a protective effect against MS (139, 140), and its protective effect is independent of HLA-DRB1*15 (140).	• The frequency of HLA-A*02 in MS patients is significantly lower than in the control group. • No significant interaction with HLA-DRB1*15, suggesting that the protective effect is independent of DRB1*15. • It has a protective effect against developing MS after EBV infection.
HLA-B*07:02	Immune response dysregulation. The HLA-B7 allele is increased in frequency in MS patients (139). The EBV-specific CD8^+^ T cells restricted by it (targeting the EBNA3A RPP epitope) exhibit functional defects, characterized by reduced secretion of IL-2, decreased expression of perforin and granzyme B, and diminished cytotoxic activity (139). Exogenous IL-2 can rescue its function, suggesting that insufficient endogenous IL-2 may be one of the mechanisms (139).	• The frequency of HLA-B7 in MS patients is significantly higher than in the control group. • Cytotoxic activity is negatively correlated with MS disease progression.
HLA-DR15	Enhance virus entry and antigen presentation. HLA-DR15 molecules can act as co-receptors for EBV entry into B cells, promoting B cell infection (141). In humanized mouse models, HLA-DRB1*15:01 is associated with poor control of EBV infection, and CD4^+^ T cells exhibit stronger cross-reactivity to EBV-infected B cells, tending to attack myelin self-antigens (142).	• It is the strongest genetic risk factor for MS. • The risk of developing MS increases more than threefold after EBV infection. • The HLA-DR15 haplotype synergizes with EBV infection, increasing the risk of autoimmunity. • Individuals carrying this haplotype are more likely to experience immune dysregulation after EBV infection.

EBV, Epstein-Barr Virus; HLA, Human Leukocyte Antigen; IFN, interferon; IL, interleukin; IM, Infectious Mononucleosis; MS, Multiple Sclerosis.

### 3.5 EBV in B cells

Following the infection of B cells by EBV, the virus induces a reprogramming of these cells, leading to the production of critical proteins, including EBNA1, EBNA2, LMP1, and LMP2. These proteins play a significant role in promoting the proliferation and immortalization of B cells.

The LMP1, encoded by the EBV, simulates the activity of CD40, which results in the activation of B cells. This mechanism enables the infected B cells to escape detection by T cell-mediated immune surveillance. The LMP1-like receptor CD40 can induce anti-apoptotic effects, thereby promoting the survival of B cells under conditions of immune stress. In addition, it promotes the proliferation of B cells by activating the NF-κB signaling pathway (90). LMP2 functions by simulating the BCR and obstructing its typical signal transduction pathways, effectively circumventing the supportive role of T cells. This is crucial for the preservation of the latent infection associated with the EBV (91). EBV-infected B cells exhibit mature BCR and IgG without undergoing the selection process in germinal centers. This attribute enables B cell clones that react to self-antigens to persist (92).

As a key protein in the replication and inheritance of the EBV epigenome, EBNA1’s unique replication mechanism serves as the core guarantee for EBV to establish and maintain long-term latent infection within B cells (93). It significantly alters the phenotypic and functional characteristics of host B cells by regulating their gene expression patterns and signal transduction pathways, providing crucial support for the survival of B cells under EBV infection (93). Additionally, the low-expression trait of EBNA1 enables it to effectively evade recognition and detection by CD8+ cytotoxic T cells (93), an immune escape mechanism that directly facilitates the persistent existence of EBV-infected B cells in the body (93).

EBNA2 is a key transcription factor for maintaining latent infection (latency III) of EBV. A prior investigation indicated that the administration of the EBNA2 inhibitor (EBNA2-TAT) has a substantial impact on the expression of genes associated with MS risk, specifically TRAF3, CD40, CLECL1, TNFAIP8, and TNFRSF1A, following the inhibition of EBNA2. This finding demonstrated that EBNA2 may engage with these MS risk genes, potentially facilitating the survival and immortalization of B cells. In addition, EBNA2 can also regulate LMP1 and LMP2, facilitating the expansion and persistence of B cells infected with EBV (94).

Of note, EBV can also evade immune surveillance by regulating the immune response (**Figure 3b**). EBNA1 has the capacity to stimulate the expression of CXCL12 while simultaneously downregulating NKG2D ligands, which facilitates the recruitment of regulatory T cells in response to natural killer (NK) cell activity. EBNA2 can transcriptionally activate a range of immune regulatory genes, such as TNF, LT-α, IL-18R, and PD-L1, while simultaneously suppressing the expression of interferon response genes and HLA II. Furthermore, the microRNAs encoded by EBV can target the host’s immune-related genes, including critical components of the type I IFN signaling pathway. This targeting impairs the immune response, leading to the attenuation of CD8+ T lymphocyte activity. Separately, EBV employs viral proteins to achieve a similar immunosuppressive effect. For instance, it produces BNLF2a to inhibit antigen presentation and exploits the host’s own immunosuppressive cytokine, IL-10, by encoding a viral homolog (vIL-10). These multifaceted strategies are critically important for establishing and maintaining EBV latency within the host (95, 96).

## 4 Clinical treatment for EBV-associated MS

**Figure 4:**
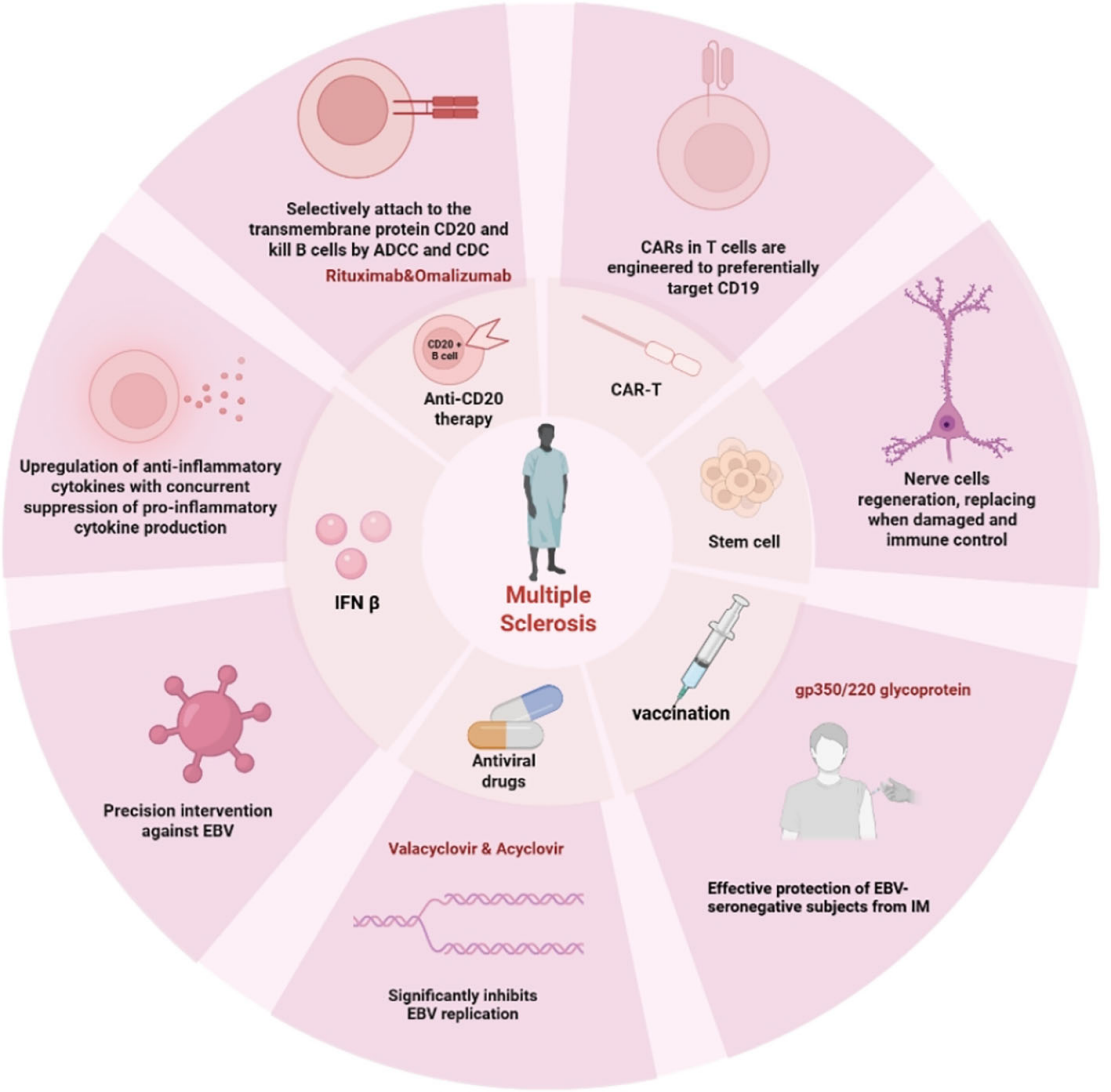
Pharmacological targets for multiple sclerosis. Several treatment options include IFN β, Anti-CD20 therapy, CD-19 CAR-T, stem cell, antiviral drugs, and vaccination, as well as their pharmacological targets. This figure is illustrated by Biorender.

In this section, we will summarize emerging methods for EBV-associated MS treatment based on supporting molecular mechanisms discussed in Section 3. Associated treatments are shown in **Figure 4**.

### 4.1 Disease-modifying therapies

Recombinant IFN β has been used for many years and was the first immunotherapy approved for the treatment of MS (97). However, in recent years, it has been replaced by other emerging drugs. The role of IFN-1β in the management of MS has been reviewed elsewhere (98–100). In brief, IFN β can increase the concentrations of anti-inflammatory cytokines, specifically interleukin-4 (IL-4) and IL-10, while simultaneously suppressing the synthesis of pro-inflammatory cytokines, including tumor necrosis factor-alpha (TNF-α) and interleukin-17 (IL-17). Meanwhile, it can downregulate the expression of MHC II on antigen-presenting cells, regulate the activity of T cells, and reduce the infiltration of inflammatory cells in the CNS. Notably, research has shown that IFN β can directly target EBV. It can interfere with the replication of EBV, inhibit the expression of its early genes, and reduce the number of EBV-specific TCRs, thereby attenuating EBV-specific immune responses in pwMS (101, 102). However, the efficacy of IFN β is modest compared to newer therapies, and long-term use may induce neutralizing antibodies that reduce its clinical benefit (103). These limitations have largely relegated IFN β to a second-line option in current practice.

In contrast to IFNβ, due to the significant role of EBV-infected autoreactive B cells in the pathogenesis of MS, CD20 inhibitors are very effective in relapsing MS, possibly because of their ability to interfere with the EBV life cycle and immune response (104). Currently, the most extensively used anti-CD20 monoclonal antibodies (mAbs) worldwide include Ocrelizumab and Rituximab, which have both high efficacy and safety in past clinical trials (105–107). Anti-CD20 treatment is highly effective in inflammatory disease in MS, at least partially by depleting the body’s aberrant proliferative B cells, but it comes with a long-term pharmaceutical requirement. Meanwhile, anti-CD20 therapy also causes normal B cells to be depleted, impairing the immune function and increasing the risk of opportunistic infection (108). At the same time, it is worth noting that this anti-CD20 monoclonal antibody therapy primarily targets CD20-expressing B cells, including pre-B cells, immature B cells, naïve B cells, and memory B cells. Intriguingly, a small subset of T cells also express CD20 (109, 110). These CD20+ T cells exhibit a pro-inflammatory phenotype, produce higher levels of IFN-γ, IL-17, and TNF-α compared to their CD20-counterparts, and are found in the cerebrospinal fluid and brain lesions of pwMS. Importantly, anti-CD20 therapy effectively depletes these CD20+ T cells, which may contribute to its therapeutic efficacy in MS (109, 110). Could this lead to immunosuppression and consequently cause EBV reactivation? Most studies have shown that the recurrence rate of pwMS remains at a very low level after monoclonal antibody treatment. However, some studies have mentioned that after discontinuing the medication, clinical and MRI subclinical activities of pwMS may recur (111). This may be because fluctuations in immune function can induce the reactivation of EBV, which further leads to the recurrence of MS. At present, further research is still needed in this field. In particular, whether mAb treatment can induce the recurrence of MS and how to reduce such recurrence risks by optimizing treatment regimens have become important topics of clinical and scientific concern.

Despite the efficacy of these immunomodulatory therapies, most of the current clinical drugs are aimed at reducing the inflammatory response in the CNS. However, none have demonstrated strong evidence towards neuron repair and remyelination or a significant effect on progression. In a phase III trial, after 12 weeks of treatment, pwMS were observed to have slightly lower disease progression rates and MRI activity after Ocrelizumab than placebo. However, during the 24-week-long-term surveillance, the results showed that the effect of monoclonal antibodies on key clinical progression was consistent with observations at 12 weeks (112). This trial also included patients with active demyelinating disease, so it is unclear if the benefit applies to patients without evidence of active disease.

### 4.2 EBV targeted therapies

In addition to teriflunomide mentioned above, which has direct anti-EBV activity, some antiviral drugs also have a direct inhibitory effect on EBV. For instance, approved antiviral drugs, such as acyclovir and valacyclovir, can significantly inhibit EBV replication (113–115). Yet, clinical trials have not clearly shown that these drugs have an obvious effect on the progression of neurological deficits (116). The reasons for this limited effectiveness may include the fact that most drugs struggle to effectively cross the BBB and fail to reach concentrations high enough to suppress EBV replication within the CNS (117, 118). Additionally, as previously noted, EBV primarily contributes to disease in MS through immune-mediated processes rather than direct viral replication, so merely inhibiting viral replication might not be sufficient to fully change the disease’s progression. Thus, the potential mechanism for MS still needs further exploration.

Vaccination has a proven track record of successfully eradicating numerous virus-associated diseases, which positions it as a promising intervention for the prevention of MS associated with EBV infection. The envelope protein of EBV has emerged as the main target of EBV-targeting vaccines, such as gp350, gB, and gH/gL, among others (20, 119–121). In addition, the latest research showed that R9AP is an important receptor for EBV to invade epithelial cells and B cells, which can directly bind to the EBV glycoprotein gH/gL complex, and can be used as a potential target for EBV prevention and vaccine development (122).

Clinical trial results further validate the potential of the EBV vaccine in preventing related diseases and activating specific immune responses, providing key data support for its subsequent application in the prevention and treatment of MS (**Table 3**). In 2007, Sokal EM et al. conducted a phase II clinical trial to confirm the effectiveness of the recombinant gp350 vaccine in protecting EBV seronegative subjects from IM. After receiving 3 doses of vaccine, the development of IM after EBV infection can be prevented to some extent (121). This study first demonstrated the efficacy of the EBV vaccine in related diseases. In 2023, a retrospective cohort study suggests that early exposure to EBV in individuals with siblings may help prevent the development of MS, prompting the potential function of EBV vaccination in early adolescence to eliminate MS (52). Unfortunately, there are still no EBV vaccines that are officially approved, proven to be safe and effective in the long term, and can be routinely put into clinical use. However, in November 2025, West China Hospital of Sichuan University completed the world’s first clinical application of an investigational EBV vaccine, with no adverse reactions reported to date. This breakthrough also reflects that the progress of EBV vaccine development is showing promising prospects. What’s more, multiple vaccines are in the development stage, and some clinical trials are ongoing (e.g., NCT06735248, NCT06908096, NCT05164094, and NCT07478952). Although vaccines may not entirely eliminate the risk of EBV infection, there is considerable optimism regarding the prevention and management of the associated diseases.

**Table 3:** Clinical trials and results of EBV-related vaccines.

Vaccine	Clinical trail	Outcomes	Reference
Recombinant gp350 vaccine	A Phase II study	• The vaccine induced a seroconversion rate of 98.7% in EBV-seronegative subjects, and anti-gp350 antibodies could persist for more than 18 months. • The vaccine has a prevention efficacy of 78% against IM. • This vaccine failed to prevent asymptomatic EBV infection. • The vaccine is safe, and its reactogenicity is acceptable.	(121)
Recombinant gp350 vaccine	Phase I/II studies	• The vaccine has confirmed immunogenicity in healthy adults and can induce gp350-specific antibody responses. • The vaccine is safe, and its reactogenicity is acceptable.	(143)

DCs, Dendritic Cells; EBV, Epstein-Barr Virus; IM, Infectious Mononucleosis.

Despite the extensive potential for the development of an EBV vaccine, several critical challenges remain to be resolved, including the optimal age for vaccination, the target population, and whether the vaccine prevents EBV infection itself or only IM. Additionally, it is uncertain whether the vaccine can reverse existing EBV-related autoimmune reactions or provide therapeutic benefits for individuals already diagnosed with MS. The answers to these questions will define how EBV vaccines can best be used for MS in the future.

Overall, the current treatment strategies for MS mainly focus on inhibiting the inflammatory response in the CNS. However, there is a lack of therapies that encourage myelin repair or nerve regeneration. Almost all immunomodulatory therapies face common challenges: the safety of long-term medication use, disease activity rebound after discontinuation, and limited efficacy for progressive MS. In addition, existing therapies are mostly based on empirical immune modulation. Although some therapies can indirectly affect EBV-infected B cells, there is still no therapy directly targeting EBV-related specific pathogenic mechanisms that has been approved for clinical use. Future research should focus on developing new drugs with neuroregenerative effects, optimizing personalized treatment strategies to balance efficacy and risk, and exploring therapies that directly target EBV or EBV-specific immune responses, such as EBV vaccines and EBV-specific CAR-T cell therapy.

### 4.3 CAR-T cell therapy

Looking beyond established DMTs and EBV targeted therapies, a new treatment based on genetic engineering technology, chimeric antigen receptors (CAR) -T cells, has brought new hope to the treatment of MS. CD-19 CAR-Ts have been engineered to preferentially target CD-19 in immune-mediated illnesses to decrease B cells more efficiently in a non-major histocompatibility complex (MHC)-restricted way (123). Although there is a lack of relevant clinical trial data, substantial progress has been made in animal experiments. In the treatment of mouse models of autoimmune encephalitis, both anti-CD19 CAR-T cells and anti-DC1 CAR-T cells demonstrated efficacy in alleviating symptoms through the reduction of lymphocyte infiltration. These results provide a foundation for future clinical investigations (124, 125). In addition, a recent case report indicated that in two pwMS who received CD19 CAR-T cell infusion, no immune effector cell-associated neurotoxicity syndrome occurred, showing good short-term tolerance and safety. Research has demonstrated that CAR-T cells can cross the BBB and expand in the CSF. Notably, one patient exhibited a marked reduction in intrathecal antibody production and oligoclonal bands, while the other patient, despite no observed reduction in oligoclonal bands, demonstrated 27-fold enrichment of CAR-T cells in the CSF in the absence of any neurotoxic symptoms (126). These preliminary clinical findings provide an important rationale for the application of CAR-T cell therapy in MS, while also revealing considerable inter-individual variability in treatment response, as not all patients achieved favorable biomarker improvement.

Of note, the expression profiles of CD19 and CD20 on B cells differ substantially. CD20 expression is largely confined to the stages from pre-B cells to memory B cells, while CD19 displays a wider range of expression, covering plasmablasts and certain plasma cell populations (127, 128). As the predominant effector B cell subset in the CSF of pwMS, plasmablasts are present throughout the disease course, and their numbers strongly correlate with both intrathecal IgG synthesis and inflammatory disease activity detected by MRI (129, 130). Due to the absence of CD20 expression on these plasmablasts, traditional anti-CD20 monoclonal antibodies are unable to eliminate them, thereby allowing the continuous production of pathogenic antibodies within the CNS. Thus, CD19/BCMA-targeted CAR-T cell therapy may offer distinct advantages over CD20-targeted approaches. By eliminating pathogenic plasmablasts, it could more comprehensively inhibit intrathecal antibody production and potentially even achieve the conversion of oligoclonal bands to negativity. This mechanism explains why CAR-T therapy can still demonstrate significant clinical efficacy in refractory MS patients who do not respond to anti-CD20 monoclonal antibody treatment.

Despite the considerable promise of CAR-T cell therapy in MS, its clinical application is confronted with multiple challenges. Persistent B cell aplasia and hypogammaglobulinemia are well-recognized as long-term complications of CAR-T therapy (131, 132). However, these data are derived primarily from patients with B-cell malignancies, and the prolonged safety profile of CAR-T therapy in MS patients remains to be further investigated. Second, the personalized manufacturing process makes CAR-T therapy costly and limits its widespread application. Third, current clinical evidence for CAR-T therapy in MS is mainly based on small case reports and phase I trials, with a conspicuous lack of large-scale phase III clinical trial data to validate its efficacy and safety. Thus, despite the theoretical advantage of CD19 CAR-T therapy in achieving more comprehensive B cell depletion, whether this potential can be translated into superior clinical outcomes compared to anti-CD20 monoclonal antibodies in real-world practice awaits confirmation through head-to-head comparative studies.

## 5 Conclusion

In this review, we have discussed the possible relationship between EBV infection and MS and potential pathogenic mechanisms based on current and past knowledge.

For patients, precise and prompt diagnosis is of paramount importance. Considering the possible association between EBV infection and MS may enhance the efficiency of patients’ diagnosis. We highlight the significance of indirect immune-mediated mechanisms, including molecular mimicry and neuroinflammation, in the pathogenesis of MS linked to EBV infection. Among these, the EBNA1-GlialCAM axis is currently supported by the most direct evidence, including clonally expanded B cells in the cerebrospinal fluid, large-scale cohort validation, and structural biology analyses, whereas ANO2 and CRYAB, though also implicated, have relatively less direct evidence. Further, alleles associated with genetic susceptibility, such as HLA DR-15, may elicit a heightened and intense T-cell response, thereby intensifying the consequences of EBV infection. Nonetheless, additional molecular studies are required to explore the precise mechanisms that contribute to the development of MS following EBV infection.

Current advancements in MS treatment are being informed by a novel understanding of the mechanisms through which EBV infection may be associated with the development of MS. In conjunction with established conventional therapies, we have investigated several novel strategies that have been the focus of research in recent years, including antiviral medications, vaccination approaches, and CAR-T cell therapy. However, it is noteworthy that though prophylactic and therapeutic vaccines hold significant potential for pwMS, they are still in the early stages of clinical translation and clinical trials. Consequently, there is an urgent need for additional research to facilitate their development. As described, although many studies provide evidence for the association between EBV infection and MS, further research is necessary to validate this relationship and elucidate the underlying mechanisms, which are essential for the development of novel therapeutic interventions.
